# Impact of High Hydrostatic Pressure, Ultrasound, and Pulsed Electric Field in Beverages Fermentation: A Review of Nutritional, Functional, and Sensory Aspects and the Future

**DOI:** 10.3390/foods14203576

**Published:** 2025-10-21

**Authors:** Sebastián Pizarro-Oteíza, Fernando Salazar, Romina Cea, Oscar Cavieres, Maninder Meenu

**Affiliations:** 1 Laboratorio de Fermentaciones Industriales, Escuela de Alimentos, Facultad de Ciencias Agronómicas y de los Alimentos, Pontificia Universidad Católica de Valparaíso, Av. Waddington 716, Valparaíso 2340000, Chile; fernando.salazar@pucv.cl (F.S.); romicear72@gmail.com (R.C.); oscar.cavieres@pucv.cl (O.C.); 2 National Agri-Food Biotechnology Institute (NABI), Mohali 140308, India; meenu_maninder@yahoo.com

**Keywords:** emerging technologies, fermented beverages, bioactive compounds, sensorial attributes, probiotic and prebiotic properties

## Abstract

This review evaluated the impact of emerging non-thermal technologies, such as high hydrostatic pressure (HHP), ultrasound (US), and pulsed electric fields (PEF), on the properties of fermented beverages. According to the information gathered, HHP improves cellular integrity and antioxidant activity of beverages, while US enhances nutrient release and improves the nutritional profile by increasing peptide content and cell viability. For its part, PEF accelerates fermentation and enhances the accumulation of bioactive compounds, which improves the sensory quality of the product. Despite their potential, the industrial implementation of these technologies faces several challenges, including technical limitations, economic constraints, and issues related to consumer acceptance. Consequently, further research is required to optimize processing parameters and address these obstacles. Overall, these technologies represent a promising approach for developing high-quality fermented beverages that meet the growing demand for health benefits and environmental sustainability.

## 1. Introduction

Fermentation is a processing and preservation technology that occurs both spontaneously and in a controlled form and varies based on raw materials, microorganisms, and processing conditions [1,2,3]. This technology is based on the use of the metabolic pathways of microorganisms to stabilize, transform, and enhance the value of products with bioactive compounds, pigments, and other bioproducts [4,5]. Conventionally, various starter microorganisms are used, including yeasts, molds, alkaline bacteria, acetic acid bacteria, and lactic acid bacteria [1,6]. The proper selection of these starter microorganisms reduces variations in sensory, nutritional, and rheological properties, in addition to inhibiting the development of spoilage-causing species [3,7].

**Figure 1:**
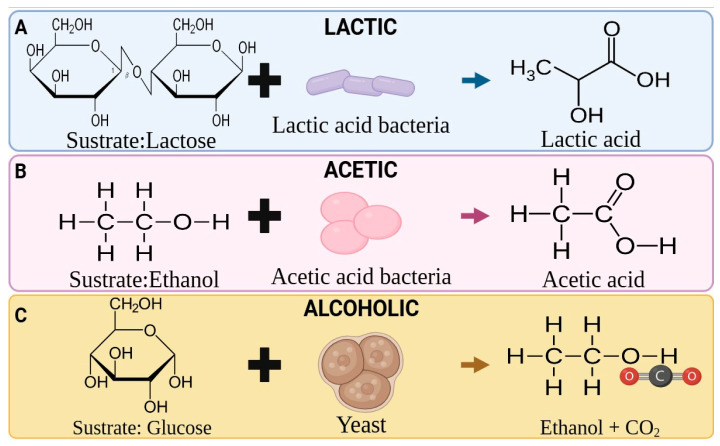
Substrates, Starter Cultures, and common products in Fermenting Beverages production. (**A**): Lactic Fermentation, (**B**): Acetic Fermentation and (**C**): Alcoholic fermentation.

Fermented beverages (FB) are liquid foods prepared mainly from fruits, vegetables, cereals, algae, dairy products or derivatives, and other matrices with microorganisms involved under suitable conditions. It is also possible to use the waste from different food industries to prepare FB [3,8]. This liquid matrix goes through a fermentation process, where microorganisms break down the substrate into simpler molecules such as alcohols, organic acids, and other products illustrated in Figure 1 [6,9,10]. Fruits and vegetables contribute functionality to FB through phenolic compounds, affecting sensory, nutritional, and antimicrobial properties [11,12]. Likewise, those made from cereals are characterized by complex flavor profiles, attributed to the formation of alcohols, organic acids, and aldehydes [13]. On the other hand, dairy-based products are characterized by sensory properties such as taste, aroma, and consistency [4]. Similarly, the potential for the use of algae in the production of FB is considerable [14]. The use of algae brings different benefits to the quality of the fermented beverage, such as the high prebiotic potential. In their production process, they usually have conventional stages such as mashing or saccharification, which aim to extract compounds from the initial substrate to form the must to be fermented [15]. The conventional way of FB production cannot achieve maximum profit from the processing sources. The increase in temperature and treatment time causes the loss of thermolabile compounds, mainly bioactive compounds, reducing the functional and sensorial quality [16]. The implementation of modern technologies has reduced operating time and energy costs. This has resulted in higher yields and more stable, high-quality products. In turn, fermentation is positioned as a more efficient process in terms of production and environmentally friendly [8,17].

Emerging technologies such as ultrasound (US), high hydrostatic pressure (HHP), and pulsed electric fields (PEF) could contribute to an improvement in the nutritional, sensory, and functional quality of the fermented beverage. In recent years, studies have proposed the combination of US, HPP, and PEF to facilitate beverage fermentation to preserve or increase the content of bioactive compounds while increasing the fermentation rate. In addition, to maintain and improve the physicochemical and sensory properties compared to the fermented beverage produced by a conventional process [8,18,19]. The change in sensory properties generally presented a positive response from the consumer towards the final product [8,20]. 

Furthermore, the application of these technologies as a pretreatment or simultaneously with fermentation shows an impact on the behavior and growth of microorganisms, the bioavailability of nutrients, the increase in bioactive compounds, prebiotic properties, probiotics, and antioxidant capacity, among others [21].

Likewise, it manages to influence physicochemical parameters such as pH or total acidity and an increase in alcohol yield [22,23,24]. The final products are of high nutritional and functional quality, being considered functional foods because they can contribute to the health of consumers, mainly attributed to their prebiotic, probiotic, antioxidant, anti-inflammatory, anticholesterolemic, and antidepressant properties. The health benefits of FB consumption are related to the digestive, immune, cardiovascular, and mental systems, mainly provided by bioactive compounds [11,12,25,26]. This review critically explores the influence of emerging processing technologies such as high hydrostatic pressure, ultrasound, and pulsed electric fields on the production and quality of fermented beverages from diverse raw materials. It also addresses their effects on nutritional, functional, sensory, and physicochemical properties and discusses current challenges, technological limitations, and prospects for industrial application.

## 2. Types of Fermented Beverages

### 2.1. Fruits and Vegetables

Fruits and vegetables have approximately 70–90% water in their composition. The largest solid component is carbohydrates, including starch, glucose, and fructose, which are higher in fruits than vegetables. In addition, fruits and vegetables are appreciated for their higher mineral content (potassium, magnesium, and calcium), vitamins, and bioactive compounds [12,27,28]. Fruit and vegetable juice is the most widely used substrate for FB production due to its composition. It contains different organic acids, such as malic or citric acid, which provide sensory characteristics accepted by the consumer [12]. FB made from fruits and vegetables is often considered a functional beverage owing to the high number of phytochemicals available in the final product, such as vitamins, phenols, and pigments, which also contribute towards antioxidant, anti-inflammatory, anticholesterolemic, and other characteristics [12,29]. The ripened, healthy fruits and vegetables are used to prepare FB in different forms.

The juice or puree form, which is cloudy or translucent, allows greater availability of sugars and nutrients, enhancing the fermentation process. These food matrices have a high fermentable sugar content; sometimes no additional sugar is required. In juice or puree forms, mainly alcoholic, acetic, or lactic-type fermentations are carried out using *Saccharomyces* spp., *Acetobacter* spp., *Lactobacillus* spp., or *Pediococcus* spp., respectively [30,31,32]. The main examples are wine, vinegar, and cider, as well as fruits used for the brewing of beers and kefirs [10,12,27,33,34,35,36].

Similarly, FB can also be prepared from horticultural products that are not fruits, such as edible roots, leaves, and stems, in addition to tubers. These substrates are characterized by their high starch or cellulose content, which can be transformed into simpler sugar units, together with high vitamin A, C, and E content, pigments such as carotene or betalain, and phenols [12,20,37,38].

In this case, fermentation is mainly conducted by LAB; the most used species is *Lactiplantibacillus plantarum*. The examples of FB made from these raw materials are agave ferments for the production of tequila, potato ferments for the production of vodka, and sugar juice ferments for the production of cachaça [39].

### 2.2. Cereals

It is also possible to prepare FB with two different types of grains: cereals are the seeds of plants belonging to the Gramineae family, such as wheat, maize, rice, barley, oats, rye, and millet, among others. On the other hand, quinoa and chia are considered pseudo cereals [40]. Generally, cereals have a high content of complex carbohydrates, mainly starch. They are also notable for the presence of significant amounts of protein, fiber, and vitamins [20]. Fermentation of cereals improves the nutritional and sensory properties of resultant FB, and they stand out for their high carbohydrate, protein, and fiber content [1,13,20]. The realization of cereal-based FB is generally accomplished by LAB, which ferments the available sugars previously obtained from polysaccharides through the action of endogenous or exogenous enzymes. This type of fermentation improves the nutritional and sensory properties of FB by generating new aromas, flavors, and consistencies [13,40,41].

### 2.3. Herbs

Herbs, according to their place of origin, species, and variety, have a diverse nutritional and phytochemical composition, with water being predominant in some cases and followed by carbohydrates in others. Most of them have a high mineral content (potassium, calcium, magnesium, and iron), as well as bioactive compounds such as catechins, theanine, caffeine, quercetin, menthol, menthone, limonene, carvone (mint), or alpha, beta, and xanthohumol acids (hops) [42,43,44]. These compounds mainly provide antioxidant, anti-inflammatory, antimicrobial, anticarcinogenic, and cardiovascular protection and improved brain function, among others [15,44,45]. In addition, the herbs can be used in FBs in two typical forms. One as a raw material; the main example of this type is kombucha, which is made from black and green tea leaves (*Camellia sinensis*) and sugar by fermenting with a combination of endogenous or inoculated LAB microorganisms and osmophilic yeasts, called SCOBY [16,45,46]. The second form of using herbs is as a secondary ingredient in other FBs, such as the addition of peppermint (*Mentha* spp.), thyme (*Thymus* spp.), oregano (*Origanum* spp.), lavender (*Lavandula* spp.), chamomile (*Matricaria chamomilla*), sage (*Salvia* spp.), lemongrass (*Cymbopogon citratus*), moringa (*Moringa oleifera*), turmeric (*Curcuma longa*), hops (*Humulus lupulus*), and rosemary (*Rosmarinus officinalis*) in the production of beers, wines, and ciders, conferring floral flavor and aromas, in addition to bioactive compounds typical of the herb type [15,44,45,46,47,48]. The resultant FB is characterized by low alcohol content, carbonation, and being a good source of organic acids [49].

### 2.4. Dairy Products

Fermented beverages made from dairy products are an important part of the food industry. Mainly milk, regardless of its fat content, milk powder, and whey protein isolate are used [50,51]. They are characterized by their high protein content, with casein and whey proteins standing out. Their main fermentable sugar is lactose. It also has a high proportion of minerals, which promote fermentation [2,4]. Generally, lactic fermentations are carried out by inoculating or allowing yeasts, kefir granules, and mainly LAB to act [4,5]. These microorganisms change the pH of the medium and provide the product with probiotic and sensory properties such as consistency (creaminess), organic acids, and longer shelf life, as well as conferring beneficial effects such as improved digestion and gut function [52,53,54].

### 2.5. Seaweed

The production of FB based on seaweed responds to the challenge of considering new alternatives for obtaining nutrients for human consumption [25].

The nutritional composition of seaweeds is notable for its high content of protein, polyunsaturated fatty acids, fiber, vitamins, and minerals [14,55]. The extraction of fermentable sugars is challenging due to the high presence of complex polysaccharides, such as alginate. Therefore, enzymatic saccharification is used as a pretreatment in the production of BF [56]. It also stands out for its bioactive compounds, such as polyphenols, sterols, alkaloids, flavonoids, and tannins [55]. Seaweeds are a suitable raw material for producing FBs, such as *Gracilaria fisheri* fermented by *L. plantarum* DW3 which provides probiotic functional properties [57]. Another example is the enzymatic protein hydrolysates of red algae (*Porphyra* spp.), new bioactive peptides with amylase inhibitory potential, and used LAB incubated at 50 °C for 24 h [58].

On the other hand, the hypolipidemic effects of *Laminaria japonica*-based beverages fermented by *S. cerevisiae* and *L. plantarum*, which showed high biliary acid binding capacity, a significant lipase inhibitory effect against pancreatic lipase, and potential to alleviate lipoprotein metabolism disorders [59].

### 2.6. Waste Products

In the search for alternative and sustainable sources to produce FB, the waste products or by-products derived from different raw materials have also been explored [9]. FB based on agricultural waste is mainly composed of lignin, cellulose, and hemicellulose from the non-consumable or usable portion, which can be further broken down to monosaccharides through various processes [60]. Various authors have pointed out that pineapple peel corresponds to approximately 29–50% of solid waste and has a high sugar content (over 8.2%), high fiber content, enzymes, and phenolic compounds in addition to their high concentration of carbon and nitrogen [61,62,63]. Thus, pineapple is a fermentable substrate and is established as a viable and sustainable alternative for producing FBs [62,64].

## 3. Fermented Beverage Processing

### 3.1. Conventional Method

Traditionally, FBs are differentiated according to their composition, microorganisms involved, and brewing processes (Table 1), and the processing of the different FBs involves similar stages. For example, before fermentation, the substrate is prepared to obtain and increase the amount of those molecules necessary for fermentation. In the case of beer, the mashing stage aims to obtain fermentable sugars in the wort by immersing the raw material (malt) in water at 65–67 °C [65]. Similarly, during kombucha production, tea leaves are infused to obtain bioactive compounds from the tea leaves (tannins) and to dissolve the added sugar [15,44,66]. For red wine maceration, the juice with fermentable sugars was obtained by pressing and left to settle with the grape skins to obtain compounds responsible for coloring.

This can be done at 10–16 °C, or in a much cooler environment, close to 4 °C [67]. Whereas, in the case of white wine, contact with the skins is not needed or is very short, and the processing temperatures were approximately 18 °C [68]. FB production is based on the transformation of substrates into value-added compounds by microorganisms. To optimize this process, enzymatic saccharification, a technique that involves breaking down polysaccharides into simpler fermentable sugars, is used [44,65]. Traditionally, raw materials are used to produce FB. These substrates already contain their own enzymes that initiate the hydrolysis process. For example, cereal grains possess amylases, which can break down starch into sugars. However, to achieve more efficient and controlled saccharification, exogenous enzymes, specific to a particular raw material and production target, are often used [15].

These exogenous enzymes act during maceration, a stage when substrate for fermentation is extracted. The use of exogenous enzymes increases fermentation yield by ensuring higher availability of fermentable sugars. That, in turn, improves beverage quality by influencing the final flavor and aroma profile and reduces processing times by accelerating the conversion of polysaccharides into sugars [69,70].

Different enzymes such as cellulase, α-amylase, β-glucosidase, xylanase, β-glucanase, and pectinase are applied during maceration [28,40,71]. In the case of kombucha production, enzymes such as polyphenol oxidase or tannase are used to increase the total phenol content in FB [70]. 

Furthermore, the fermentation process requires the presence of microorganisms, which traditionally are obtained either endogenously (substrates/environment) or exogenously (specific strains); both ways are called starter culture.

**Table 1:** Conventional Processing Methods for Fermented Beverages.

Fermented Beverage	Source	Fermentation Conditions	Microorganisms	References
Beers	barley, malted barley, wheat, rice, rye, corn, oats, sorghum, bread	Lager 7–21 days; 5–15 °C; pH 4.0–5.5 Ale: 2–6 weeks;18–27 °C; pH 4.0–5.0 Lambic: 3–9 months; 15–25 °C; pH 3.8–4.4	*Saccharomyces* spp., *Brettanomyces* spp., *Pediococcus* spp., *Hanseniaspora* spp., *Saccharomyces* spp., *Brettanomyces* spp., *Pediococcus* spp., *Hanseniaspora* spp., *Lactobacillus* spp., *Acetobacter* spp. and *Gluconobacter* spp.	[34,72,73]
Fruit beers	blueberries, cherries, peaches, raspberries, strawberries, mangoes, apples, pears, pineapple, banana			
Free gluten beers	rice, corn, quinoa, buckwheat, amaranth, oats, and sorghum			
Wine	grapes	Alcoholic: 10–15 days; 14–30 °C; pH 4.5–6.5 Malolactic: 2–12 weeks; 20–30 °C; pH 3.5–6.5	*Saccharomyces* spp., *Lactobacillus* spp., *Leuconostoc* spp., *Oenococcus* spp., and *Pediococcus* spp.	[33,36]
Fruit wine	blackberry, pineapple, passion fruit, banana, and watermelon			[27,35]
Rice wine	rice	First fermentation: 5–14 days; 15–30 °C; pH 4.5–5.5 Second fermentation: 1–2 months; 10–15 °C; pH 4.0–5.0	*Saccharomyces* spp., *Mucor* spp., *Bacillus* spp., *Aspergillus* spp., *Rhizopus* spp. and *Lactobacillus* spp.	[74]
Vinegar	blueberry, persimmon, sugar cane, beer, citron, plum, dates, pomegranate, grains, soursop, cherry, kombucha, malt, mango, apple, molasses, honey, rice must, orange, pears, pineapple and its by-products, banana and papaya, whey, grape, wine	Alcoholic: 7–14 days; 18–20 °C; pH ~5.0 Acetic: 1–2 weeks; 25–40 °C; pH 5.5–7.0	*Saccharomyces* spp., *Acetobacter* spp.	[34,75]
Cider	apple	First fermentation: 1–4 weeks; 15–30 °C; pH 3.5–6.5 Second fermentation: 1–3 weeks; 10–17 °C; pH 3.5–5.0	*Saccharomyces* spp., *Hanseniaspora* spp., *Torulaspora* spp.	[22,34]
Kefir	milk, water, sugar, nuts, herbs	1–4 days; 18–30 °C; pH 2.5–7.0	*Lactobacillus* spp.	[10]
Kombucha	sugar, black tea and green tea	1–3 weeks; 20–30 °C; pH 3.5–4.5	*Komagataeibacter* spp., *Gluconobacter* spp., *Acetobacter* spp., *Brettanomyces* spp.	[15,44,45,76,77]
Kvass	Rye, malt, sugar, water	1–2 weeks; 18–25 °C; pH 3.5–4.5	*Lactobacillus* spp., *Leuconostoc* spp., *Saccharomyces* spp., *Brettanomyces* spp.	[48]
Yogurt	milk	4–8 h; 40–45 °C; pH 4.3–4.6	*Streptococcus* spp., *Lactobacillus* spp., *Bifidobacterium* spp.	[4,50,78]
Seaweed fermented	brown, red and green algae	50 °C for 24 h	*Lactobacillus* spp., *Saccharomyces* spp., *Lactiplantibacillus* spp.	[57,58,59]

Different strains of microorganisms can be used depending on the fermentation type [2]. The starters mostly used are *S. cerevisiae* for alcoholic fermentation, LAB like *Lactobacillus* spp. or *Streptococcus* spp. for lactic fermentation, and AAB like *Acetobacter* spp. for acetic fermentation [6,10].

The application of different strains during fermentation develops a variety of sensorial profiles, besides avoiding a fermentation stop or the development of spoilage-causing, pathogenic, and competitor microorganisms [3,5].

### 3.2. Emerging Technologies

The production of fermented products has progressed considerably over time, from natural fermentation to controlled industrial processes. That involved various innovative methods for improving the nutritional, functional, and sensory quality of the resultant products [79,80]. In recent decades, the metabolism of microorganisms and their role in fermentation have been studied, in addition to new procedures and the application of emerging technologies to increase process efficiency [81].

**Figure 2:**
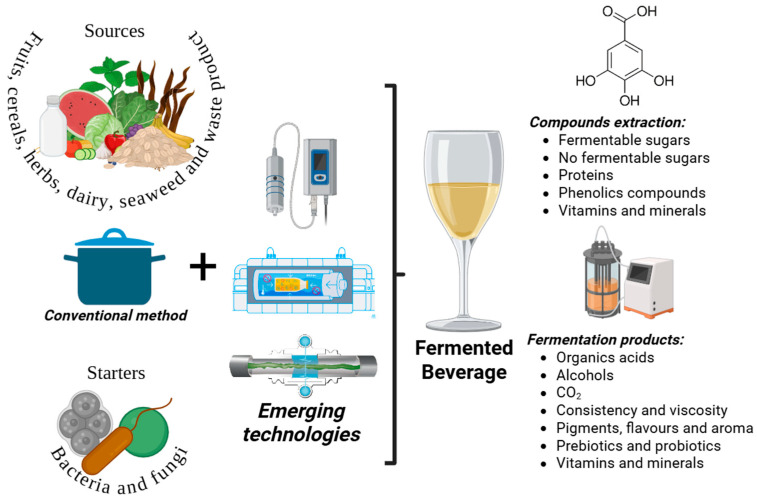
Overview of Fermenting Beverages Production Assisted by Emerging Technologies.

Ultrasound (US), high hydrostatic pressure (HHP), and pulsed electric fields (PEF) are some of the emerging technologies employed for this purpose [8,48,82]; see Figure 2. The use of emerging non-thermal technologies (<40 °C) can accelerate chemical reactions, reduce processing time, improve organoleptic properties, improve the quality of FB, increase fermentation yield, and extract health-promoting compounds [8,19].

#### 3.2.1. Ultrasound (US)

Ultrasound (US) is the sound waves that pass through the food matrix at frequencies higher than 20 kHz at variable power. US treatment comprises three main effects, namely, mechanical damage to the food, formation of free radicals, and heat generation [83].

**Figure 3:**
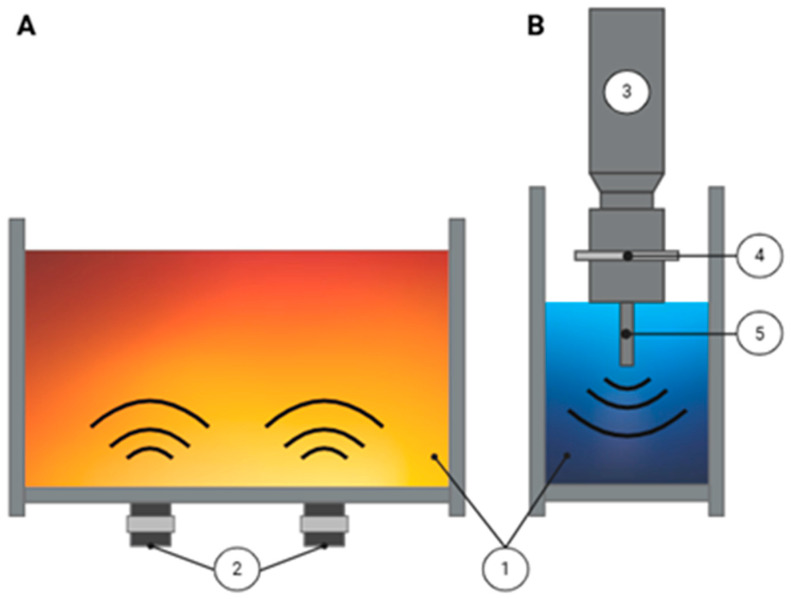
(**A**): (1): Fermenting beverages, (2): Ultrasound bath, (**B**): (1): Fermenting beverages, (3): Transducers, (4): Amplifiers, and (5): Horn.

Acoustic cavitation is the most studied phenomenon related to US treatment, which consists of the expansion and rupture of gas bubbles generated in the cells of the matrix, leading to the permeation of compounds into a liquid medium [84,85,86]; see Figure 3.

These changes promote cell permeability, the release of intracellular compounds, and the activation of enzymes. During fermentation, the combination of high power (>1 W) and low frequencies (20–100 kHz) of US stimulates the growth and metabolism of microorganisms due to the enhanced permeability of cells that promotes the transfer of nutrients and metabolic residues across the membrane [84,87].

Depending on the intensity of application, it is possible to improve the extraction of phenolic compounds and reduce fermentation time, and depending on the strain used, the hydrolysis of sugars can also be increased or decreased.

The use of ultrasound (US) has been shown to enhance enzymatic activity involved in the degradation and transformation of compounds during fermentation [4,18]. At high frequencies above 100 MHz and low power below 1 watt, US is mainly applied for analytical monitoring of fermentation processes. Additionally, ultrasound induces cavitation, generating physical and biochemical changes that accelerate fermentation and improve the extraction of bioactive compounds. This dual role makes US treatment a valuable tool both for process analysis and intensification. Among cavitation methods, hydrodynamic cavitation stands out as a scalable and energy-efficient technology suitable for industrial applications. It is generated by passing liquids through mechanical constrictions that produce cavitation bubbles, which disrupt vegetable cell walls and enhance mass transfer in continuous, solvent-free processes. 

Hydrodynamic cavitation has shown significant benefits in extracting phenolic compounds, sugars, and essential oils and in biomass pretreatment for biofuel production [88].

#### 3.2.2. High Hydrostatic Pressure (HHP)

The high hydrostatic pressure (HHP) employs elevated pressures from 100 to 1000 MPa for 5 to 15 min. HPP is a non-thermal technique employed to improve food quality and shelf life while maintaining certain quality attributes The HHP process is based on Le Chatelier’s principle and Pascal’s principle (isostatic) [89].

The first principle states that if the dynamic equilibrium is disturbed due to changes in conditions, the equilibrium position is modified to restore the equilibrium, causing the food product to produce an opposite reaction.

**Figure 4:**
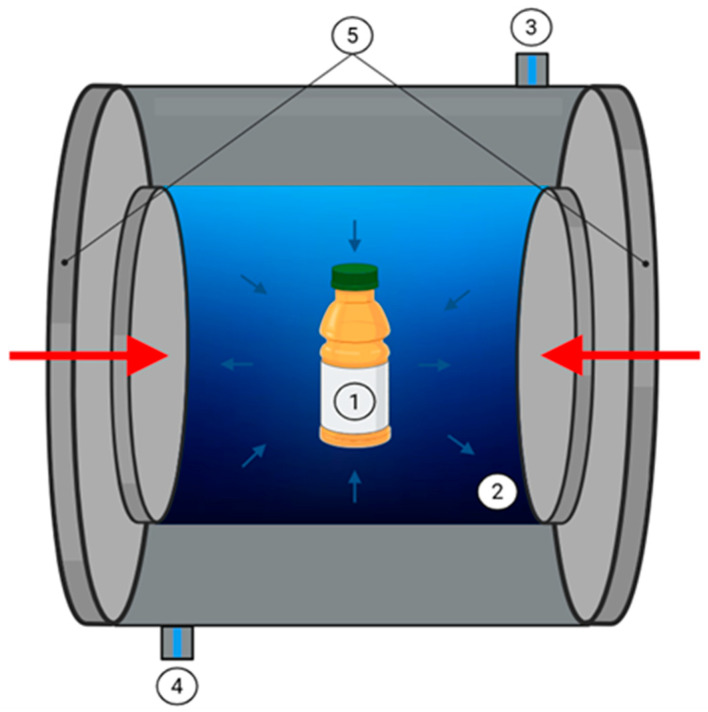
High Hydrostatic Pressure Equipment: 1. Fermenting beverage; 2. Pressure-transmitting fluid; 3–4. Liquid inlet and outlet; 5. End closures. Note: Arrows on the system (left and right) according to Pascal’s principle.

As for Pascal’s principle, it is postulated that pressure is transmitted uniformly to the food in all directions, and after decompression, the food returns to its original shape [89] see Figure 4. The main effect of HHP is an alteration of protein structures, which enhances dough properties [90]. In addition, HHP enhances the quality and safety of FB by improving structural integrity, fermentation performance, and bacterial strain survival. It preserves original organoleptic properties, increases nutritional value, and enhances antioxidant activity [90].

#### 3.2.3. Pulsed Electric Fields (PEF)

**Figure 5:**
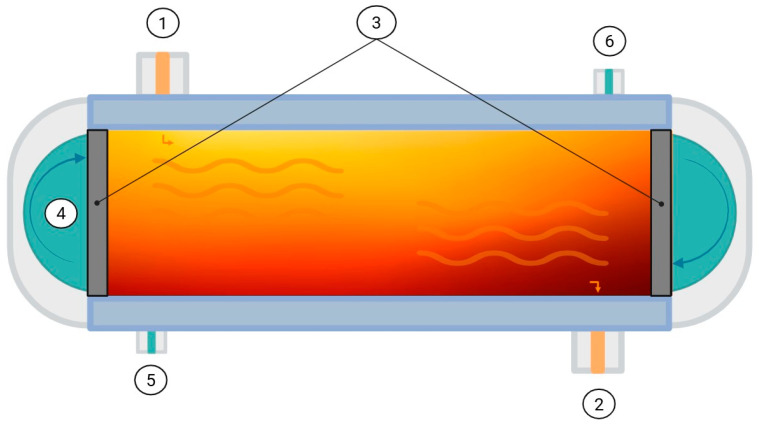
Pulsed Electric Fields Equipment: 1–2. Fermented beverage inlet and outlet; 3. Electrodes; 4. Water jacket; 5–6. Water inlet and outlet.

Pulsed electric field (PEF) technology uses short, high-voltage pulses ranging from 1 to 40 kV/cm, which are applied to solid, semi-solid, or liquid foods through two electrodes for brief durations ranging from microseconds to a few minutes, depending on the type of cells and the characteristics of the medium [8,22,91] (see Figure 5). In addition, depending on the intensity of the treatment, electroporation may be either reversible or irreversible, each leading to different effects during fermentation.

In some cases, this enhances the process without negatively impacting other product characteristics, as the membrane pores may close over time.

Under specific experimental conditions, PEF can function either as a microbial growth inhibitor or as a promoter, while also improving the extraction of bioactive compounds [22,86,92]. Furthermore, the application of electrical pulses to microbial cells through PEF enhances the exchange of intracellular and extracellular substances. This process can accelerate fermentation, modify metabolite production, and regulate microbial growth. Its effectiveness depends on several factors, including the intensity of the electric field, pulse duration, the characteristics of the food matrix, and the specific microorganisms involved [82].

## 4. HHP, US, and PEF in Fermented Beverage Production

### 4.1. Effects on Microorganisms

In the production of fermented beverages, maintaining a balanced microbial ecosystem is essential to ensure product safety, as well as desirable sensory and functional properties. Although heat treatments have traditionally been employed to stabilize the microbiota, they can negatively impact heat-sensitive compounds and beneficial microorganisms. As an alternative, non-thermal technologies such as high-pressure processing (HPP), pulsed electric fields (PEF), and high-intensity ultrasound (US) have emerged as promising tools that enable selective modulation of the microbiota without compromising nutritional or sensory quality [93,94,95]. These technologies act through distinct physical mechanisms: PEF induces electroporation, disrupting cell membranes; US generates cavitation, leading to mechanical damage; and HPP applies isostatic pressure that denatures intracellular structures [96].

For instance, US treatment (20 kHz, 3 W/L, 11.09 min) has been reported to increase viable *Lactobacillus rhamnosus* counts by 40.11% [85]. Similarly, US has been shown to reduce the fermentation time of *Lactobacillus acidophilus* in sweet whey by approximately 30 min [97]. These findings highlight the potential of non-thermal technologies to enhance fermentation efficiency and microbiological safety, particularly in sensitive products such as kombucha, liquid yogurt, and low-sulfite wines. However, the use of high ultrasound amplitudes may lead to microbial inactivation and reduced fermentative activity, likely due to decreased extracellular enzyme concentrations [85,98,99]. Despite these limitations, US has proven to be an effective tool in the development and optimization of fermented beverages.

High hydrostatic pressure (HHP) treatment (200–400 MPa for 10 min) has also demonstrated positive effects, improving antioxidant capacity, pH stability, and cell viability in apple juice prior to fermentation. These conditions facilitated nutrient release and increased the availability of binding sites for *L. plantarum*. After 24 h of fermentation, levels of caffeic, ferric, and chlorogenic acids increased, suggesting that HHP could be a potential alternative to pasteurization for enhancing fermented apple juice quality. Furthermore, *L. plantarum* demonstrated a survival rate of 97.37% in simulated gastric fluid post-fermentation [96].

In another study, HHP-assisted fermentation of yacon–litchi–longan juice (300–500 MPa, 25 °C, 15 min) inoculated with *Gluconacetobacter xylinus* and *L. rhamnosus* enhanced protein content, promoted the formation of volatile flavor compounds, and increased ketone concentration in the final product [100].

Pulsed electric field (PEF) treatment has shown similar benefits. Applied to yogurt starter cultures, field strengths varied between 1 and 3 kV/cm (50–150 Hz, 4–8 μs pulse duration, 50 pulses); this treatment reduced yogurt fermentation time from 5 h to 4.18 h [50]. PEF application to apple juice (285 V/cm, 1.4 cm electrode distance) both before and after fermentation led to a 25% increase in biomass concentration and a 45% increase when the inoculum was pretreated [22]. Additionally, PEF (1 V/cm, 60 Hz, 2–5–40 h) enhanced bacteriocin production by *L. acidophilus* during fermentation without affecting the lag [24]. The summary is shown in Table 2.

Table 2, evaluated the impact of various assisted technologies on microbial behavior during fermentation processes, taking into account different substrates, microorganisms, and treatment conditions. The technologies investigated include ultrasound (US), high hydrostatic pressure (HHP), and pulsed electric fields (PEF).

In dairy substrates such as whey and milk, ultrasound exhibited several beneficial effects, including the preservation of viable cell counts, enhancement of metabolic activity, reduction in fermentation times, and increased production of biomass and metabolites. Furthermore, in buffer solutions, US was shown to improve enzymatic activities (notably β-galactosidase), increase lactic acid yield, and enhance cell permeability. 

Similarly, in plant-based beverages and fruit juices, ultrasound facilitated ethanol release and contributed to shorter fermentation durations.

HHP led to a partial reduction in fermentable sugars in fruit juices, while simultaneously ensuring high survival rates of probiotic microorganisms under simulated gastrointestinal conditions. Additionally, HHP treatment resulted in increased levels of bioactive compounds, thereby potentially enhancing the nutritional value of the products.

Moreover, the use of pulsed electric fields in buffer solutions, juices, and dairy products significantly promoted bacteriocin production, increased biomass, and notably shortened fermentation times. PEF also modulated microbial viability and reduced ethanol production in certain strains, demonstrating its versatility in fermentation process optimization.

Therefore, these assisted technologies represent powerful tools for enhancing fermentation processes by improving microbial viability, biomass and metabolite yields, and reducing overall fermentation time. Consequently, their application holds great promise for improving the quality and functional properties of fermented food products.

### 4.2. Nutritional and Functional Properties

During the last decades, different approaches have been developed to better understand microbial metabolism and its role in fermentation. In this sense, the application of emerging technologies could improve the functional and nutritional properties of FB and increase the speed of biochemical processes or generate changes in the metabolic pathways of the different microorganisms involved in processing [102]. According to previous studies, consumption of FB has demonstrated different benefits due to their probiotic, prebiotic, antioxidant, anti-inflammatory, and nutritional properties, specifically micronutrients.

Examples of these benefits are improving digestion, bone, and mental health; strengthening the immune system; and reducing the risk of cardiovascular disease and certain types of cancer. Various matrices have been studied to enhance their health benefits by incorporating bioactive compounds and nutraceuticals [29,103].

Recent studies have shown that antioxidants, phenolic compounds, and flavonoids in fermented beverages help prevent cell damage, aging, and serious diseases such as cancer, coronary heart disease, and cerebrovascular disease [104,105]. In addition, the intake of probiotic and fermented foods can be beneficial for many gastrointestinal diseases and also exhibit anti-pathogenic, anti-allergic, anti-angiogenic, anti-cancer, anti-inflammatory, anti-diabetic, and anti-obesity effects, and even positive effects on the brain and central nervous system [25,106]. On the other hand, there is a great challenge for the industry to guarantee consumer demands, such as different formats, delivering beneficial properties for consumer health, and maintaining the high nutritional value used [21,28,103]. In the case of proteins, fermentation can lead to improved digestibility, as proteins are partially hydrolyzed into polypeptides, oligopeptides, and free amino acids, making them more accessible and easier to absorb in the human body [17].

PEF, US, and HHP have been mainly used as microbiological inactivation alternatives in FB due to their ability to inactivate bacteria and yeasts. However, it has been demonstrated that, according to the treatment conditions, favorable changes can be induced during fermentation to maintain or improve their functional and nutritional properties, which results in enhanced consumer benefits [8,18,19,20,21]. The summary is shown in Table 3. Ultrasound treatment in yogurt caused an increase in peptide content of 28.7% and 16.1% when applied to the inoculum before fermentation and during fermentation, respectively. US treatment for 35 min with a power of 100 W/L and a frequency of 28 kHz increased peptide content by 64.23%. This improved the functional properties, such as the prebiotic and probiotic content of the resultant product [98].

On the other hand, the fermentation of sweet whey inoculated with microorganisms previously treated with the US (20 kHz, 84 W, 150 s) presented an increase in the number of viable cells in a logarithmic cycle over the conventional heat treatment (37 °C/30 min) [97]. Increasing the amplitude and application time of the US resulted in a higher number of viable cells. This finding was attributed to the deagglomeration of bacterial colonies and the formation of pores in the cell membranes that increase their permeability and modify the chemical affinity between the substrate and the enzymes [7,99].

The increase in live cells in FBs is essential for health, thanks to the presence of probiotics and prebiotics. Probiotics are living microorganisms that support digestion, strengthen the immune system, and maintain a balanced gut flora [103]. Furthermore, they function as nutrients for the microorganisms in the human gut, promoting their development and function. This collaboration between probiotics and prebiotics supports a healthy gut microbiota, which is crucial for preventing disease and enhancing quality of life. This indicates that the consumption of fermented beverages (FB) with a high percentage of live cells can improve both gut and overall health. As reported, FBs are recognized by the International Scientific Association for Probiotics and Prebiotics (ISAPP), which defines them as foods produced through microbial growth and enzymatic conversion of components [102].

Recent studies have shown that treating a fermented almond beverage with high-intensity ultrasound (20 kHz, 450 W, 6 min, 25 °C) prior to inoculation does not affect its metabolic activity. However, when the ultrasound treatment is applied before the addition of probiotics, the stress conditions generated during fermentation enhance the survival of microorganisms during storage [23]. Ultrasound has also been considered a promising tool for accelerating fermentation and enhancing the functionality of whey- and oat-based beverages without compromising their quality [107]. In contrast, fermentation of apple juice treated with high hydrostatic pressure (HHP) resulted in significantly higher total phenol content compared to pasteurized apple juice during the pre-fermentation stage. The increased phenolic content suggests an enhanced potential for antihyperglycemic and antihyperlipidemic effects in the fermented product [96]. Similarly, applying high hydrostatic pressure (HHP) at 100–300 MPa or 600–700 MPa to milk intended for yogurt production resulted in higher amino acid content in the final product. Therefore, HHP pretreatment of milk could be a promising strategy to improve the nutritional quality of yogurt [108].

On the other hand, ref. [109] applied PEF to Merlot grapes (up to 41.5 kV/cm and 49.4 kJ/kg), achieving greater extraction of anthocyanins and phenols such as catechin, as well as a reduction in 2-hexenal (green aroma). After fermentation, the wines had higher levels of anthocyanins, stilbenoids, and phenolic acids. Reference [110] applied PEF (5 kV/cm, 48 kJ/kg) to skins and must, using 100 monopolar pulses of 10 μs at 0.5 Hz. The treatment significantly increased phenolic compounds in the freshly fermented model wine, with increases of 48% in flavonols and 18% in total phenols, exceeding those achieved with enzymes.

Emerging non-thermal technologies such as US, HHP, and PEF have proven to be effective in preserving and improving the nutritional properties of fermented beverages. HHP stands out for preserving phenolic compounds, antioxidant capacity, and probiotic viability [96,111]. PEF maintains bioactive compounds without affecting the nutritional profile [112,113], while US favors nutrient release and bioavailability during fermentation to improve quality in fermented dairy products and wines [114,115].

**Table 3:** Effects of US, PEF and HHP technologies on Fermented Beverage properties.

Assisted Technology	FB	Fermented Beverage Properties	Reference
US	Mixed kefir	Increase antioxidant activity by 28% (22 kHz, 90 W/L, 3 min) Improved sensory properties (22 kHz, 90 W/L, 3 min)	[83]
	Yogurt fermenter	Increase vitamin C content by 28% (22 kHz, 120 W/L, 3 min) Increase antioxidant activity by 67% (22 kHz, 90 W/L, 3 min)	
	Industrial kefir	Increase vitamin C content by 30% (22 kHz, 120 W/L, 3 min) Increase antioxidant activity by 67% (22 kHz, 90 W/L, 3 min)	
	Active peptide yogurt	Increases peptide content (28 kHz, 100 W/L, 35 min)	[98]
	Tepache	Maintains titratable acidity and pH (25 kHz, 20–100%, 5–10 min) Promotes changes in the microstructure and composition (25 kHz, 20–100%, 5–10 min)	[84]
	Chinese rice wine	Decreases sugar content (28 kHz, 35 W/L, 1 h, seventh day) Increases total acid content and content of ester (28 kHz, 35 W/L, 1 h, seventh day) Maintains pH value (28 kHz, 35 W/L, 1 h, seventh day)	[5]
	White millet beverage	Increases total phenol content and antioxidant activity (20 kHz, 3 W/L, 40.11%, 11.09 min) Increases flavonoid content (20 kHz, 3 W/L, 41.42%, 2.63 min) Decreases particle size (20 kHz, 3 W/L, 41.42%, 2.63 min)	[85]
HPP	Yogurt	Increases pH level (700 MPa, 10 min) Decreases total solids content (500–600–700 MPa, 10 min) Enhanced firmness (700 MPa, 10 min) Decreases wheying off level (500–600–700 MPa, 10 min) Improves sensory properties like color, flavors, taste, and firmness (700 MPa, 10 min)	[108]
	Yacon-Litchi-Longan juice	Decreases 13.75% of free amino acids content (500 MPa, 25 °C, 15 min) Loss of 3.67% of total volatile flavors compounds (500 MPa, 25 °C, 15 min)	[100]
	Shalgam	Maintains pH and total soluble solids (100–500 MPa, 20–40 °C, 5–15 min)	[93]
	Apple Juice	Increases caffeic, ferric, and chlorogenic acid levels after 24 h (200 MPa, 10 min) Decreases pH level (200 MPa, 10 min) Decreases color an 80% (300 MPa, 10 min) Increases total phenol content (200 MPa, 10 min) Maintains antioxidant activity (200–300–400 MPa, 10 min)	[96]
PEF	Yogurt	Slightly decreases in pH (1 kV/cm, 4 Hz, 50 pulses) Fastest decreases in oxidation reduction potential (3.67 kV/cm, 0.5 Hz, 50 pulses)	[101]
	Yogurt	Decreases pH (1 kV/cm, 150 Hz, 8μs, 400μs, 3.8 J)	[50]
	Wines	Up to 41.5 kV/cm, 49.4 kJ/kg. improved the extraction of anthocyanins and phenols such as catechin, as well as reducing 2-hexenal. Increases in total phenol content (8 kV/cm, 344 Hz, 300 s)	[109,115]

These technologies constitute a promising alternative to conventional thermal methods for the development of fermented beverages with improved nutritional value.

### 4.3. Organoleptic and Physicochemical Characteristics

In fermented beverages, pH, color, and sensory attributes are crucial factors for ensuring microbiological safety, visual quality, and a satisfactory consumer experience. Reference [50] reported that cultures treated with PEF (50 monopolar pulses with an electric field intensity between 1 and 3 kV/cm and a frequency between 50 and 150 Hz) underwent faster acidification during the first three hours of fermentation, as evidenced by a greater drop in pH compared to the control. This effect was attributed to the early stimulation of *Streptococcus thermophilus*, a species more sensitive to PEF than *Lactobacillus bulgaricus*. Based on the above, pulsed electric field (PEF) treatment is understood to promote this phenomenon, which is attributed to reversible electroporation induced by low-intensity fields. This process enhances nutrient uptake and accelerates microbial metabolism, resulting in increased lactic acid production by lactic acid bacteria (LAB) [50]. On the other hand, similarly, fruit-based kombuchas exhibiting a measured “yellow-amber” color are associated with fruity descriptors according to consumer panels [116]. Likewise, a fermented yogurt made from potatoes and blueberries, with a pH close to 4.5 and a distinct color profile, demonstrated a smooth sensory perception characterized by low acidity, bitterness, and astringency [117]. These findings underscore that coordinated control of pH and color not only ensures product safety but also directly influences texture, flavor, and consumer acceptance. In addition, ref. [109] analyzed that a sensory perspective, Merlot grapes with PEF intensities of up to 41.5 kV/cm and energies of up to 49.4 kJ/kg stood out for their more intense blackcurrant aroma and flavor, as well as differences in spice flavor.

The application of nonthermal technologies such as high hydrostatic pressure (HHP), ultrasound (US), and pulsed electric fields (PEF) in fermented beverages facilitates the preservation and even enhancement of critical parameters, including pH, color, and sensory attributes. For example, in fruit juices like strawberry juice, combined treatment with PEF and high-power ultrasound (HPU) stabilizes pH values while maintaining chromatic coordinates within acceptable ranges without compromising pigment integrity [118]. In rosé wine, ultrasound treatment has been shown to enhance color intensity and increase phenolic and aromatic compound concentrations without elevating astringency [119]. Furthermore, the combined application of HHP (450 MPa for 5 min) and ultrasound (5 min at 600 and 1200 W/L) preserves pH and acidity, retains over 90% of organic acids, and increases anthocyanin content by up to 24%, without significant changes in instrumental color, soluble solids, or sensory perception [119]. Thus, these nonthermal technologies offer effective alternatives for optimizing physicochemical and sensory quality in fermented beverages without compromising safety or organoleptic properties.

Ultrasound, HHP, and PEF treatments have demonstrated efficacy in preserving physicochemical and sensory attributes of fermented beverages, including color, taste, and texture [23,97,120]. Sensory parameters such as appearance, color, and aroma are critical quality determinants in shalgam, a fermented turnip-based beverage. Notably, HHP application does not significantly degrade these attributes. Analyses of key colorimetric parameters showed no statistically significant changes in the red hue of shalgam following HHP treatment [121]. Similarly, fermented pomegranate juice treated with HHP at 300 and 400 MPa for durations ranging from 2 to 25 min-maintained color stability [122].

Although research on the sensory effects of emerging technologies such as US, HHP, and PEF in fermented products remains limited, recent studies highlight their promising potential. HHP treatment of kimchi juice, a traditional Korean fermented vegetable beverage, effectively preserved color stability and volatile flavor compounds without compromising microbiological safety [123]. These sensory improvements correlated with physicochemical changes, including increased total phenolics and flavonoids, contributing to enhanced antioxidant activity [124]. Moreover, ultrasound processing increased organic acid concentrations, adding complexity to the vinegar’s flavor profile.

## 5. Future Perspectives, Challenges and Opportunities

Over the past decade, non-thermal technologies such as high hydrostatic pressure (HHP), ultrasound (US), and pulsed electric fields (PEF) have emerged as promising alternatives to conventional thermal treatments in the production of fermented foods and beverages. These methods offer significant advantages, including enhanced microbiological safety and improved product quality, while preserving the nutritional and sensory attributes of the final product. Despite these benefits, the industrial implementation of such technologies remains limited due to a combination of technical, economic, and regulatory challenges.

In the case of PEF, its efficacy in enhancing fermentation processes has been widely documented. However, the technology faces technical barriers such as electrode corrosion, bubble formation leading to non-uniform treatment, and the potential for metallic contamination. These limitations are further compounded by high initial investment costs, the absence of standardized processing parameters, and regulatory hurdles. In many regions, PEF-treated products require additional classifications, such as GRAS status or designation as “novel foods,” which slows down their path to commercialization [125,126].

Similarly, the application of HHP faces obstacles related to scalability, energy consumption, and equipment limitations. The need for high-capacity, pressure-resistant vessels results in substantial capital investment, which explains the relatively low adoption rate of around 18% among large-scale producers and even lower penetration among small and medium enterprises [127].

Despite these challenges, recent evidence highlights the potential of PEF as a pretreatment for improving various aspects of fermentation. In winemaking, continuous application of PEF at field strengths of 1.2–1.6 kV/cm has been shown to enhance the extraction of phenolic compounds without negatively impacting sensory attributes [128]. In non-alcoholic lactic fermentations, the use of PEF in a recirculating system preserved the viability of *Lactobacillus delbrueckii* and resulted in acidification kinetics comparable to untreated controls [129]. Furthermore, PEF has demonstrated the ability to improve the extraction of anthocyanins and phenolic compounds, reduce maceration time and temperature, promote mannoprotein release, accelerate aging processes, and lower the need for sulfur dioxide (SO_2_), thus positioning itself as a valuable tool in modern oenology [130]. While these findings underscore the technological potential of PEF for industrial-scale applications, further research is needed to fully understand its mechanisms of action across diverse fermentation substrates [8].

US treatment is another non-thermal technology with considerable promise, particularly due to its capacity to enhance extraction efficiency, preservation, and drying. However, its industrial adoption is constrained by several factors. Technical limitations stem from the heterogeneity of food matrices and the lack of standardized parameters for frequency, intensity, and transducer configuration, which hinders reproducibility and scalability [131]. The economic feasibility of ultrasound systems is also a concern, given their high initial costs, ongoing maintenance requirements, and the periodic replacement of transducers. Moreover, the localized nature of ultrasonic waves necessitates the use of multiple transducers or complex multi-stage setups to ensure uniform treatment, further increasing operational complexity [132]. Regulatory gaps add another layer of difficulty, as neither the FDA nor EFSA has established specific guidelines regarding the use, labeling, or safety of ultrasound-treated foods, creating uncertainty for manufacturers [133]. Furthermore, acoustic cavitation, driven by high-frequency ultrasound, is effective at small scales and for heat-sensitive compounds but faces limitations in industrial scalability due to energy distribution issues, higher costs, and potential thermal degradation. Therefore, hydrodynamic cavitation is considered a more practical and cost-effective solution for large-scale extraction processes, while acoustic cavitation is better suited for precise, small-batch applications [88].

Consumer perception represents an additional barrier to the broader adoption of emerging non-thermal technologies. Skepticism about unfamiliar processing methods, such as PEF and US, can negatively influence purchasing decisions. In this regard, targeted communication strategies that clearly articulate the safety, environmental benefits, and technological reliability of these treatments are essential to improve acceptance and market penetration. Simultaneously, additional research is needed to address critical gaps, including the long-term physicochemical and sensory stability of treated products, techno-economic evaluations, and the operational scalability of these systems under continuous industrial conditions.

One of the most promising strategies for overcoming the limitations of individual non-thermal technologies lies in their combined use. Synergistic applications of PEF, US, and HHP have demonstrated improved outcomes in terms of microbial inactivation, compound extraction, and product stability.

For example, the combined application of PEF and US to a milk–orange juice beverage led to a 50% reduction in ochratoxin A (OTA) and a 47% reduction in enniatin B (ENNB), outperforming individual treatments in complex dairy-fruit matrices [134]. Likewise, the co-application of HHP and PEF has been shown to enhance microbial destruction when used in conjunction with bioactive compounds [135]. These findings are supported by broader evidence indicating that PEF, US, and HHP contribute to improved process efficiency, reduced degradation of bioactive components, and lower environmental impact compared to conventional thermal methods [136,137].

Ultimately, the future success of non-thermal technologies in fermented beverage production will depend on progress in four key areas: the standardization of technological parameters, the economic viability of industrial systems, the harmonization of international regulatory frameworks, and the development of consumer trust. Achieving sustainable and large-scale integration of these technologies will require a multidisciplinary approach that includes energy optimization, modular equipment design, life cycle assessments, regulatory clarity, and collaborative business models. As research continues to evolve, the integration of non-thermal technologies into mainstream production may redefine the future of fermented beverage innovation.

## 6. Conclusions

Emerging non-thermal technologies such as ultrasound (US), high hydrostatic pressure (HHP), and pulsed electric fields (PEF) have demonstrated significant potential to optimize fermentation processes through distinct yet complementary physical mechanisms. Ultrasound generates acoustic cavitation, increasing cell membrane permeability, stimulating microbial activity, accelerating biochemical reactions, and enhancing the extraction of bioactive compounds. High hydrostatic pressure modulates the fermentative microbiota by inactivating undesirable microorganisms without compromising sensory properties, thereby improving product safety and quality. Pulsed electric fields induce electroporation of cell membranes, facilitating nutrient release and boosting microbial metabolism, which intensifies fermentation and enriches the functional profile of beverages. Collectively, these technologies accelerate fermentation, enhance bioactive compound extraction, and reduce reliance on chemical additives. While technical and economic challenges persist for industrial-scale implementation, their optimized application can shorten fermentation time, minimize additive use, and contribute to the sustainable production of fermented beverages with superior nutritional and sensory attributes.

Scientific and technological advances have been instrumental in evolving fermentation processes, enabling the selection and optimization of microbial starter cultures and enhancing their metabolic performance through innovative processing strategies. A comprehensive understanding of the mechanisms of each technology, both individually and in combination, as well as their effects on diverse raw materials and microbial strains, is essential for defining optimal processing parameters. This approach seeks to reduce energy consumption and environmental impact while preserving the physicochemical and functional integrity of the final product. The integration of US, HHP, and PEF presents promising opportunities for the development of next-generation functional beverages that align with consumer demands for health-oriented and environmentally responsible products.

Further research is required to substantiate the potential health benefits of beverages produced through these non-thermal methods, as their efficacy depends on processing parameters, substrate properties, and microbial characteristics. Continuous optimization will be crucial to enable industrial-scale application and advance the production of safe, efficient, and high-quality fermented beverages.

Ultrasound has been shown to accelerate fermentation, enhance microbial viability, and promote the release of bioactive compounds, resulting in higher peptide and antioxidant content and improved sensory acceptance. High hydrostatic pressure applied at 200 to 700 MPa increases microbiological stability, enhances the availability of phenolic acids and amino acids, and maintains or improves organoleptic attributes such as color, flavor, and texture. Pulsed electric fields reduce fermentation time, stimulate microbial growth and metabolite production including bacteriocins, and preserve or enhance phenolic and antioxidant compounds, yielding favorable effects on sensory perception compared with conventional thermal treatments. Collectively, these emerging technologies improve the nutritional, functional, and sensory quality of fermented beverages, offering sustainable, high-value alternatives to traditional methods and highlighting their potential to transform the beverage fermentation industry.

## Figures and Tables

**Table 2:** Effects of Ultrasound (US), High Hydrostatic Pressure (HHP), and Pulsed Electric Fields (PEF) on microorganisms and Substrates used in fermented beverage processing.

Assisted Technology	Substrate	Microorganism	Effect of Treatment Conditions on Microbial Behavior	Reference
US	*Sweet whey*	*Strep. thermophilus*, *L. delbrueckii bulgaricus* and *L. acidophilus*	Minimum content of viable cells (20 kHz, 480 W, 8 min, 55 °C)	[97]
			Slightly increases pH, electrical conductivity, and viscosity (20 kHz, 480–600 W, 6.5–10 min, 45–55 °C)	
			Maintains titratable acidity (20 kHz, 480–600 W, 6.5–10 min, 45–55 °C)	
			Decreases fermentation time by 0.5 h (20 kHz, 84 W, 160 s, 55 °C)	
	*Milk*	*Lactobacillus*	Increases metabolic activity and biomass (120 and 90 W/L, 3 min)	[83]
	*Buffer solution*	*L. lactis*	Increases viable cell count and cell permeability (24 kHz, 400 W, 30%, 5 min)	[99]
			Increases β-galactosidase activity (24 kHz, 400 W, 30%, 5 min)	
			Increases lactic acid yield (24 kHz, 400 W, 20%, 5 min)	
			Decreases protein concentration (24 kHz, 400 W, 30%, 5 min)	
	*Buffer solution*	*L. brevis*	Increases viable cell count (23 kHz, 150 W, 10 μm, 5 min, 30 °C)	[7]
			Increases cell permeability and proteolysis (24 kHz, 150 W, 15 μm, 5 min, 30 °C)	
			Increases acidity of the medium (23 kHz, 150 W, 10 μm, 5 min, 30 °C)	
			Increase γ-aminobutyric acid production (23 kHz, 150 W, 10 μm, 5 min, 30 °C)	
			Decreases pH of the medium (23 kHz, 150 W, 10 μm, 5 min, 30 °C)	
	*Pineapple beverage by-product*	*S. cerevisiae*	Favors the releasing of ethanol (25 kHz, 20–100%, 5–10 min)	[84]
	*Dairy*	*S. cerevisiae*	Increases ethanol yield (28 kHz, 35 W/L, 1 h, first day)	[5]
			Decreases fermentation time (28 kHz, 35 W/L, 1 h, first day)	
	*White millet drink*	*L. rhamnosus*	Increases viable cells count (20 kHz, 3 W/L, 40.11%, 11.09 min)	[85]
			Decreases fermentation time (20 kHz, 0.83 W/L, 41.42%, 2.63 min)	
HHP	*Yacon*, *lychee* and *longan juice*	*L. rhamnosus* and *G. xylinus*	Glucose, fructose, and sucrose content was partially reduced (300–500 MPa, 25 °C, 15 min)	[100]
	*Apple juice*	*L. plantarum*	Survival of *L. plantarum* in simulated gastric fluid reached 97.37% after fermentation.	[96]
			After 24 h, caffeic, ferric, and chlorogenic acid levels increase (200–400 MPa, 10 min)	
PEF	*Buffer solution*	*L. acidophilus*	Higher bacteriocin formation (1 V/cm, 60 Hz, first 5 h, 30 °C)	[24]
			Higher biomass production (1 V/cm, 60 Hz, 2 min on/off, 37 °C)	
	*Apple juice*	*Hanseniaspora* spp.	Increases the biomass concentration by around 25% (285 V/cm, 10 pulses each 100 μs, Δt = 1 ms, Δtt = 1 s, during and after fermentation)	[22]
			Decreases ethanol content by 1.6% (285 V/cm, 10 pulses each 100 μs, Δt = 1 ms, Δtt = 1 s, during log phase)	
	*Yogurt*	*Strep. thermophilus* and *L. bulgaricus*	15.4% of the initial inoculum of *Strep. thermophilus* and 24.3% of that of *L. bulgaricus* survived (1–3.67 kV/cm, 0.5–4 Hz, 5–50 pulses)	[101]
	*Yogurt*	*Strep. thermophilus* and *L. delbrueckii bulgaricus*	Shortest fermentation time (1 kV/cm, 150 Hz, 8 μs, 400 μs, 3.8 J)	[50]

## Data Availability

No new data were created or analyzed in this study. Data sharing is not applicable to this article.

## Abbreviations

The following abbreviations are used in this manuscript:

FB	Fermented beverages
US	Ultrasounds
HHP	High hydrostatic pressures
PEF	Pulsed electric field
